# The Influence of Component Rotational Malalignment on Early Clinical Outcomes in Total Knee Arthroplasty

**DOI:** 10.7759/cureus.22444

**Published:** 2022-02-21

**Authors:** Sait Dalyan, Fırat Ozan, İbrahim Altun, Murat Kahraman, Ali Eray Günay, Koray Özdemir

**Affiliations:** 1 Orthopedics and Traumatology, Kayseri City Training and Research Hospital, Kayseri, TUR

**Keywords:** rotational alignment, total knee arthroplasty, knee, component alignment, knee pain

## Abstract

Background

The most important cause of patient dissatisfaction following total knee arthroplasty (TKA) is pain. Component rotation is an important factor in the clinical success of TKA. This study aims to determine component rotational errors in patients with mobile- and fixed-bearing polyethylene inserts after TKA and also to evaluate the effect of possible malrotations on clinical outcomes.

Methods

Seventy-five knees from sixty-six patients who underwent TKA were evaluated retrospectively. The patients were divided into two groups according to whether they received a mobile-bearing polyethylene insert (group 1, n = 48) or a fixed-bearing polyethylene insert (group 2, n = 27). The Hospital for Special Surgery (HSS) score, the Western Ontario and McMaster Universities Arthritis Index (WOMAC), the Lysholm Knee Scoring Scale, and the Oxford Knee Score were used for the clinical evaluation of the patients. The rotational state of the components was evaluated by computed tomography.

Results

The HSS, WOMAC, Lysholm, and Oxford clinical scores were not significant between the two groups (p > 0.05). The effect of femoral versus tibial component rotational deviation on clinical scores was not significant between the two groups (p > 0.05). Component rotational differences did not have a significant effect on the degree of knee flexion and extension between groups (p > 0.05). When the combined rotations of the components were compared with the clinical scores of function, no significant difference was detected between groups (p > 0.05). In addition, no significant difference between the operated sides of the patients and the combined component internal rotations was found (p > 0.05).

Conclusion

Although component rotation is an important factor in the clinical success of TKA, the current study did not find a clear association between the clinical results after TKA and the internal rotation of components. Component internal rotation alone is not an important predisposing factor for pain development after TKA. We believe that this may be attributed to the significant effects of patient expectation, which is often ignored, on clinical scores.

## Introduction

Total knee arthroplasty (TKA) is the gold standard treatment for advanced knee arthritis with satisfactory long-term outcomes [1-3]. The leading causes of TKA failure are well known and mainly include infection, aseptic loosening, instability, stiffness, polyethylene wear, and peri-prosthetic fracture [2,3]. The most important cause of patient dissatisfaction following TKA is pain [1-3]. The reported incidence of unexplained pain following TKA ranges from 8% to 19% [1,4,5]. In many cases, the location of this pain is the peri-patellar region [1].

Although the incidence of anterior knee pain is well known, its aetiology has not been clearly established [5]. Effective management of patients with unexplained painful TKA is difficult [5,6]. Technical errors in surgery, component alignment, and anterior knee pain are mostly associated with anterior knee pain [6]. Factors such as excessive varus or valgus alignment, poor prosthesis design, excessive medial or inferior patellar components, inability to maintain soft tissue balance, and rotation of the tibial and femoral components may lead to patello-femoral complications [1,4-6].

Many studies have attempted to establish a relationship between anterior knee pain after TKA and malrotations of the tibial component, the femoral component, or both [1,4-6]. Berger et al. [7] emphasised that internal rotation of the combined components is associated with patellar tilting, subluxation, and patellar component failure. Barrack et al. [6] conducted a study to evaluate the relationship between anterior knee pain and component rotation after TKA and found that patients with combined component internal rotation are over five times more likely to experience anterior knee pain after TKA compared with those with combined component external rotation (ER). The authors also reported that component malrotation is an important factor in the development of anterior knee pain after TKA [6].

The aims of this study are to determine component rotational errors in patients with mobile- and fixed-bearing polyethylene inserts after TKA and evaluate the effect of possible malrotations on clinical outcomes.

## Materials and methods

This is a retrospective study that included 75 knees (nine patients bilaterally) from 66 patients (57 [86.4%] females; 9 [13.6%] males) who underwent TKA for primary gonarthrosis were evaluated. TKA was performed on the right side in 34 (45.3%) knees and on the left side in 41 (54.7%) knees. The patients were divided into two groups according to whether they received a mobile-bearing polyethylene insert (group 1, n = 48) or a fixed-bearing polyethylene insert (group 2, n = 27). The study protocol was approved by the Kayseri Erciyes University Clinical Research Ethics Committee (Approval Date/No.: 13.11.2019/782) and conducted in accordance with the principles of the Declaration of Helsinki.

Patients who underwent posterior-stabilised cemented TKA, patients with normal component alignment in the coronal and sagittal planes on the orthoroentgenogram, patients without patellar surface replacement, patients with etiologically primary gonarthrosis, and patients who did not undergo a second surgical procedure before or after TKA surgery were included in this study. Patients with posterior-retaining or uncemented TKA, patients with post-operative knee flexion and/or extension problems, patients with varus and/or valgus misalignment, patients with periprosthetic infection, and patients with signs of aseptic loosening were excluded from the study.

The Hospital for Special Surgery (HSS) score, the Western Ontario and McMaster Universities Arthritis Index (WOMAC), the Lysholm Knee Scoring Scale, and the Oxford Knee Score were used for the clinical evaluation of the patients.

**Figure 1:**
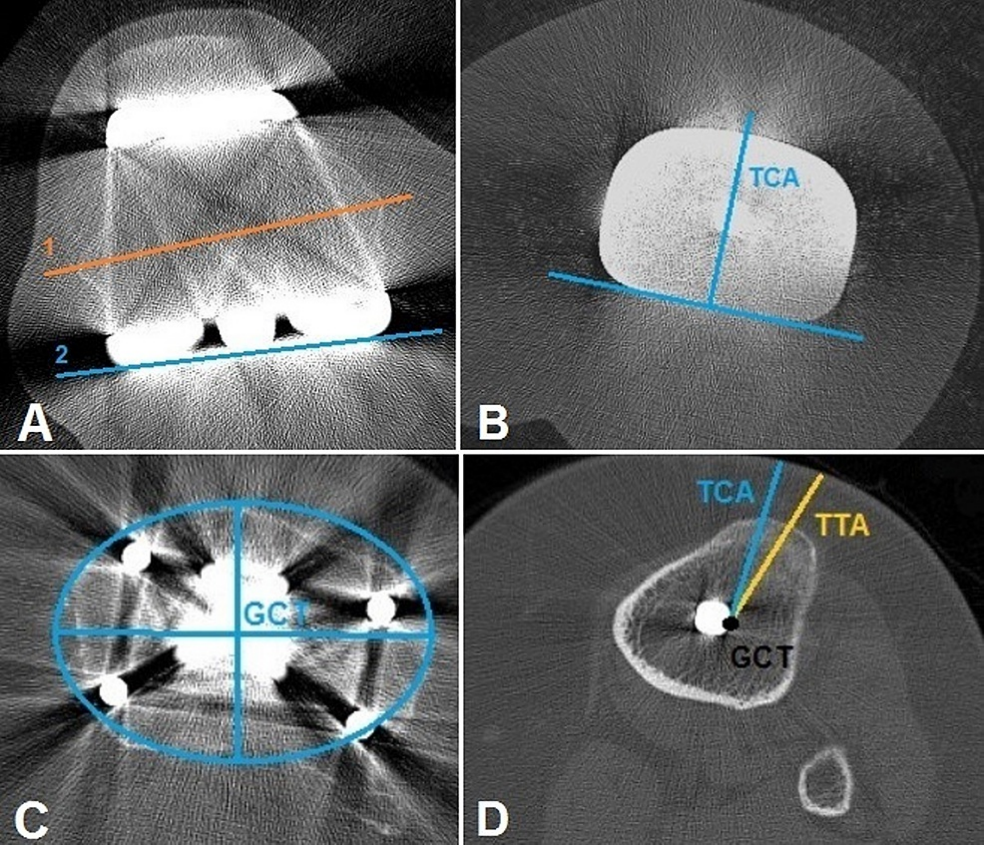
Determination of rotational alignments of the femoral and tibial components (A) The trans-epicondylar axis (1) is determined from the computed tomographic cross-section images by drawing a line from the prominence of the lateral epicondyle to the medial epicondylar sulcus. The posterior condylar axis (2) is obtained by drawing a line along the posterior rim of the implant. The angle between these two lines determines the posterior condyle angle. (B) The axis of the tibial component (TCA) is determined from the line drawn along the posterior border of the tibial component and the appearance of the line drawn perpendicular to it. (C) The geometric centre of the tibial (GCT) plateau includes the tibial plateau just below the tibial baseplate, with an ellipse inserted into the bony contours of the tibial plateau. (D) The GCT plateau is transferred to this image. The tibial tubercle axis (TTA) is determined by drawing a line from the GCT to the most prominent point of the tubercle. The angle between the TTA and the tibial component axis determines the extent of tibial component rotation.

Rotational alignments of the femoral and tibial components were calculated according to the protocol described by Berger et al. [7]. Computed tomography (CT) sections of 1.5 mm were obtained perpendicular to the components, starting from the proximal region of the femoral component and continuing to the level of the tibial tubercle [7]. Measurements were made using anatomically determined guide points from these sections, and component rotations were found. The angle between the surgical epicondylar axis and the posterior condylar axis was used whilst measuring the degree of rotation of the femoral component (Figure 1).

The degree of rotation of the femoral component was standardised by subtracting 3.5° for male patients and 0.3° for female patients from the obtained values [7]. The degree of rotation of the tibial component was measured as follows. First, a line tangent to the posterior of the tibial component and a line perpendicular to this line were drawn. Then, just below the component, the geometric midpoint of the tibial plateau was identified. This midpoint was lowered distally towards the most prominent part of the tibial tubercle (TT), and a line was drawn towards the TT. The angle between this line and the line perpendicular to the line tangent to the posterior of the tibial component was determined. Standardisation was achieved by subtracting 18° from the obtained measurement, and the degree of rotation of the tibial component was calculated.

The combined component rotation was determined by adding the rotational degrees of the femoral and tibial components. The relationship between the combined component internal rotational angle and the patello-femoral incompatibility was described as follows: a grade of 1-3° was considered mild, a grade of 4-7° was considered moderate, and a grade of 8-17° was considered severe [7]. All TKA surgeries were conducted using measured resection techniques with the intramedullary alignment of the femoral component and extramedullary alignment of the tibial component.

Statistical analysis

Statistical analysis was done using IBM SPSS Statistics 21.0 (IBM, Armonk, NY, USA), and all results were expressed as mean ± standard deviation (SD). The conformity of the data to the standard distribution was tested by using the Shapiro-Wilk test. The chi-squared test was used to analyse categorical data. Statistical significance was tested using the independent-samples t-test and one-way analysis of variance, whilst the difference between variables with a non-normal distribution was evaluated using the Mann-Whitney U test. A p-value equal to or less than 0.05 was considered significant.

## Results

The mean follow-up times of groups 1 and 2 were 1.15 ± 0.31 years (range, 1-2 years) and 1.56 ± 0.52 years (range, 1-3 years), respectively (p = 0.000). No significant difference in terms of age, side, and gender was noted between the groups (Table 1).

**Table 1:** Demographic characteristics of patients SD: standard deviation

		Group 1	Group 2	P-value
		n = 48	n = 27	
Side, n (%)	Right	23 (48)	11 (40)	0.632
	Left	25 (52)	16 (60)	
Gender, n (%)	Female	37 (86)	20 (87)	0.818
	Male	6 (14)	3 (13)	
Age, years, mean ± SD (range)		66.14 ± 6.35 (48-77)	67.91 ± 7.01 (55-81)	0.439
Follow-up time, years, mean ± SD (range)		1.15 ± 0.31 (1-2)	1.56 ± 0.52 (1-3)	0.000

No significant difference in terms of mean component rotations was observed between the two groups (Table 2).

**Table 2:** Component rotational alignment of the groups IR: internal rotation; ER: external rotation; SD: standard deviation

	Group 1	Group 2	P-value
	n = 48	n = 27	
Tibial component			
Mean ± SD	4.3° IR ± 9.43	5.6°IR ± 8.2	0.54
Range	21.7° IR-16.3° ER	20.3° IR-9.2° ER	
Femoral component			
Mean ± SD	2.38° ER ± 2.25	1.7° ER ± 2.02	0.2
Range	2.1°IR-7° ER	3° IR-5.8° ER	
Combined rotation			
Mean ± SD	1.9° IR ± 9.71	2.92° IR ± 8.14	0.65
Range	18.1° IR-17.8° ER	14.5° IR-13.3° ER	

Moreover, no significant difference in terms of HSS, WOMAC, Lysholm, or Oxford clinical scores was noted between the groups (HSS: 86.51 ± 8.11 vs. 89.57 ± 12.48, respectively, p = 0.177; WOMAC: 11.57 ± 5.36 vs. 15.83 ± 10.98, respectively, p = 0.071; Lysholm: 80.79 ± 12.66 vs. 79.52 ± 17.23, respectively, p = 0.823; Oxford: 37.39 ± 4.93 vs. 37.30 ± 7.06, respectively, p = 0.987).

The mean degree of flexion of the patients was 124.42° ± 23.33 (range, 90-150°) in group 1 and 140.30° ± 13.91 (range, 90-150°) in group 2 (p = 0.002). The mean degree of extension of the patients was 1.04° ± 2.72 (range, 0-10°) in group 1 and 0.93° ± 2.78 (range, 0-10°) in group 2 (p = 0.862).

When the femoral component rotation of the patients in group 1 was evaluated, five femoral components were found to be in internal rotation. No significant differences in the effect of femoral component rotation deviations on all clinical scores were observed in group 1 patients (Table 3).

**Table 3:** Effect of tibial and femoral component rotations on clinical scores IR: internal rotation; ER: external rotation; HSS: Hospital for Special Surgery; WOMAC: Western Ontario and McMaster Universities Arthritis Index

	Group 1						Group 2					
	n = 48						n = 27					
	Femur			Tibia			Femur			Tibia		
	IR	ER	P-value	IR	ER	P-value	IR	ER	P-value	IR	ER	P-value
	n = 5	n = 43		n = 32	n =16		n = 4	n = 23		n = 20	n = 7	
HSS Score				HSS Score			HSS Score			HSS Score		
Mean	91.60	86.60	0.190	88.06	85.25	0.255	83	91.47	0.041	91.6	86.28	0.400
(range)	(77-98)	(66-100)		(68-100)	(66-98)		(78-87)	(64-118)		(64-118)	(70-96)	
WOMAC Score				WOMAC Score			WOMAC Score			WOMAC Score		
Mean	10.60	11.54	0.702	10.78	2.76	0.211	19.5	13.91	0.576	14	16.85	0.464
(range)	(5-14)	(0-36)		(0-36)	(5-24)		(7-42)	(0-37)		(0-42)	(5-32)	
Lysholm Score				Lysholm Score			Lysholm Score			Lysholm Score		
Mean	87.60	81.07	0.270	82.91	79.44	0.367	73.25	82.34	0.576	81.15	80.57	0.607
(range)	(76-95)	(44-100)		(44-100)	(46-95)		(43-95)	(49-100)		(43-100)	(55-95)	
Oxford Score				Oxford Score			Oxford Score			Oxford Score		
Mean	40.00	38.00	0.399	38.69	37.25	0.349	38	36.86	0.622	38.35	37.71	0.685
(range)	(33-43)	(25-45)		(27-46)	(25-44)		(24-45)	(22-48)		(22-48)	(31-45)	
Flexion (°)				Flexion (°)			Flexion (°)			Flexion (°)		
Mean	130.00	123.49	0.552	124.69	123.13	0.826	142.5	100.86	0.974	143	132.85	0.808
(range)	(100-150)	(90-150)		(90-150)	(90-150)		(130-150)	(90-150)		(130-150)	(90-150)	

Femoral component rotation differences did not have a significant effect on the degree of knee flexion in group 1 patients. Whilst the mean flexional angle of knees with femoral components in the internal rotation was 130.00° ± 23.45 (range, 100-150°), the mean flexional angle of knees with femoral components in the normal rotation was 123.49° ± 22.97 (range, 90-150°; p = 0.552).

When the femoral component rotation of patients in group 2 was evaluated, four of the femoral components were found to be in internal rotation. No significant differences in the effects of femoral component rotation deviations on all clinical scores, except HSS scores, were observed in group 2 patients. Femoral component rotational differences did not have a significant effect on the degree of knee flexion in group 2 patients. Whilst the mean flexional angle of knees with femoral components in the internal rotation was 142.5° (range, 130-150°), the mean flexional angle of knees with femoral components in the normal rotation was 100.86° (range, 90-150°; p = 0.974).

When the tibial component rotation of patients in group 1 was evaluated, 32 femoral components were found to be in internal rotation. No significant differences in the effect of tibial component rotational deviation on clinical scores were observed in group 1 patients. In group 1 patients, tibial component rotational differences did not have a significant effect on the degree of knee flexion in group 1 patients. Whilst the mean flexional angle of knees with femoral components in the internal rotation was 124.69° ± 22.71 (range, 90-150°), the mean flexional angle of knees with femoral components in the normal rotation was 123.13° ± 23.86 (range, 90-150°; p = 0.826).

When the tibial component rotation of patients in group 2 was evaluated, 20 femoral components were found to be in internal rotation. No significant differences in the effect of tibial component rotational deviation on clinical scores were observed in group 2 patients. In group 2 patients, tibial component rotational differences did not have a significant effect on the degree of knee flexion. Whilst the mean flexional angle of knees with femoral components in the internal rotation was 143° (range, 130-150°), the mean flexional angle of knees with femoral components in the normal rotation was 132.85° (range, 90-150°; p = 0.808).

When the combined rotations of the components were examined, 45 patients were found to be in internal rotation whilst 30 patients were found to be in normal rotation. When the combined rotations of the components were compared with the clinical scores of function, no significant difference was detected (Table 4).

**Table 4:** Effect of combined component rotations on clinical scores IR: internal rotation; ER: external rotation; HSS: Hospital for Special Surgery; WOMAC: Western Ontario and McMaster Universities Arthritis Index; SD: standard deviation

	IR	ER	P-value
	n = 45	n = 30	
HSS Score			
Mean ± SD	88.9 ± 9.96	87.03 ± 8.89	0.424
WOMAC Score			
Mean ± SD	12.38 ± 8.06	13 ± 7.09	0.733
Lysholm Score			
Mean ± SD	81.68 ± 14.58	81.13 ± 13.03	0.869
Oxford Score			
Mean ± SD	38.4 ± 6.06	37.86 ± 5.23	0.696
Lack of extension (°)			
Mean ± SD	0.33 ± 1.65	1.72 ± 3.34	0.045

Of the components in combined internal rotation, 10 were mild, nine were moderate, and 25 were severe. No significant difference between these values and clinical scores was observed (HSS, p = 0.129; WOMAC, p = 0.588; Lysholm, p = 0.576; Oxford, p = 0.557). No significant difference between the operated side and combined component internal rotations was found (right-sided internal rotation: 18 (53%) vs. left-sided internal rotation: 27 (67.5%), p = 0.235; right-sided external rotation: 16 (47%) vs. left-sided external rotation: 13 (32.5%), p = 0.235).

## Discussion

The results of the present study do not support component malrotation as a factor contributing to the presence of anterior knee pain after TKA. Although component rotation is an important factor in the clinical success of TKA, the current study did not find a clear association between the clinical results after TKA and the internal rotation of components. This finding indicates that component internal rotation alone is not an important predisposing factor for the development of pain after TKA. In addition, no significant difference between the operated sides of the patients and the combined component internal rotations was found. We used four scoring systems to achieve a better evaluation of the effectiveness of component rotation on the clinical results.

Improvements in component design, surgical technique, and instrumentation in TKA have reduced the incidence of patello-femoral complications [1,5]. The revision rate within the first five years after primary implantation is 2.8% [1]. The importance of the rotational and axial alignments of the tibial and femoral components in knee arthroplasty is well understood [1,4-8]. In particular, component malrotation has been identified to be an important factor in pain following TKA and is associated with an increase in patello-femoral complications [1,4-9].

Many study results have highlighted the importance of component rotation in achieving successful clinical outcomes [5-7,10]. The external rotation of the femoral component relative to the posterior condyles provides optimal patellar alignment compared with neutral or internal rotation, and, unlike the internal rotation of the components, external rotation errors are better tolerated [1,5,6]. Examination of the rotational components is recommended in patients with anterior knee pain if no other cause can be found [1]. Studies have demonstrated that an internally rotated femoral or tibial component can lead to patello-femoral problems [1,5,10]. The relative internal rotation of the tibial component effectively increases the Q angle and changes the force vector on the extensor mechanism [5,6]. Abnormal stress on the patella and surrounding soft tissue may explain the observed peri-patellar symptoms [5,6].

The aetiology of anterior knee pain after TKA is often unclear [1,5]. Indeed, the causes of anterior knee pain are multi-factorial and can be divided into functional and mechanical issues [1,5,6]. In some cases, the cause of the pain may be a biological response, such as fibrous band formation, increased tension from changing kinematics, pressure on soft tissue, or component malposition [1,4,6].

The abnormal rotation of the components on CT scans is not associated with patellar tilting or subluxation on tangential radiographs [4,6,7]. Moreover, tangential radiographs are not a reliable indicator of the presence of component rotation or anterior knee pain after TKA [4,6,7]. Rotational abnormalities may cause tilting, subluxation, or excessive lateral pressure with a dynamic muscle pull that cannot be reflected on a single static radiograph [4-7]. A valid and reproducible technique using CT scans has been described to measure combined knee component rotations accurately [4-7]. In this work, we evaluated the rotational status of the components by CT scan.

Bell et al. [4] determined that, in addition to the separate excessive internal rotations of the femoral and tibial components, combined component rotations and component mismatched rotations are also factors in unexplained pain following TKA. Bell et al. [4] aimed to determine an acceptable rotational limit in TKA and found extreme internal rotational values of 5.8° in the tibial component, 3.9° in the femoral component, and 8.7° in the combined rotation. In the case of extreme external rotation, no significant difference was found between groups in terms of pain [4]. In their study of 30 patients who had undergone revision surgery for patello-femoral complications, Berger et al. [7] found that low levels of combined component internal rotations could be associated with patellar subluxation, whereas high levels of internal rotations could be associated with dislocation and component failure.

Barrack et al. [6] observed a highly significant difference in tibial component rotations between their test groups, with patients with anterior knee pain averaging 6.2° internal rotation compared with those in the control group with 0.4° external rotation. A significant difference in combined component rotations was also observed, with patients with anterior knee pain averaging 4.7° internal rotations compared with those in the control group averaging 2.6° external rotations [6]. However, no significant difference in the degree of radiographic patellar tilting or patellar subluxation was noted between the two groups [6]. Nicoll and Rowley [5] reported that at least 4.6% of their TKRs had been implanted with significant internal rotational errors and that, overall, 22 (56.4%) of the painful TKRs had internal rotational errors involving the femoral, tibial, or both components. The authors thus indicated that internal rotational errors, particularly of the tibial component, are a major cause of pain and functional deficit after TKR [5].

In our study, the maximum degree of internal rotation of the tibial component was 21.7°, whilst the maximum degree of internal rotation of the femoral component was 3°. Our findings are similar to those reported in the literature, but rotational errors in placement of the femoral component occurred less frequently and were smaller [4-6,10]. Although the association of patellar complications with component malrotation has been demonstrated by CT scanning, the relationship between malrotation and the presence of anterior knee pain is not well documented [1,5,6,8]. Therefore, component rotation is not the only cause of anterior knee pain [4-6]. Barrac et al. [6] reported that three patients with anterior knee pain in their study did not have combined component internal rotation and that five patients with combined component internal rotation did not have anterior knee pain. Consistent with these studies, no correlation between the presence of tilting or subluxation and the occurrence of anterior knee pain has been determined [11]. Similarly, Bell et al. [4] found patients in their control group who had excessive internal rotational component measurements but painless TKA. Therefore, the authors believed that the excessive rotational limits defined in their study are not absolute threshold values of acceptable rotation in TKA [4]. Excessive component rotations may be a possible factor of post-TKA pain in patients, but other factors are also involved [4].

According to Bell et al. [4], patients in the control group with internally rotated component measurements may have fewer functional demands or activity levels and, therefore, do not develop pain or score low on the functional components of clinical scoring scales. The pre-operative physiological conditions of patients are an important factor influencing whether they will eventually develop internal rotational alignment disorders [4,12-14].

Some authors suggest considering early revision if component malrotation is detected [12]. However, more well-designed studies in the field of painful rotational malrotation revision are necessary to confirm findings before this operation can be recommended as a treatment [4,13,14]. One study estimated that the rate of incompatibility of components was 4.6% [4]. In a follow-up study of patients with unexplained pain after TKA, half of the patients improved after five years of conservative treatment [13]. Therefore, conservative treatment is recommended for most patients with TKA who have unexplained pain [4].

Hadi et al. [9] reported a significant relationship between one or more malalignment parameters and functional scores in 50% of the articles they reviewed in their systemic review study. When parameters were evaluated separately in this review, 64% of the studies investigating alignment in the coronal plane did not show a relationship between malalignment and functional scores [9]. Moreover, 100% of the studies reviewed did not show an association between sagittal dissonance and functional scores [9]. Finally, 50% of the studies found a relationship between component rotational mismatch and functional scores [9].

This study has some limitations. First, this research is a retrospective study. Second, we do not have data regarding pre-operative patient expectations, which we believe may affect the post-study clinical results. This information should be taken into consideration in future studies with larger cases in this area.

## Conclusions

In our study, when femoral and tibial component rotations were evaluated in TKA patients with mobile-bearing and fixed-bearing polyethylene inserts, we found that these values did not result in a significant difference in terms of their effects on clinical scores when compared with the targeted standard rotational degrees. No clinical difference was observed between patients with mobile-bearing polyethylene inserts and those with fixed-bearing polyethylene inserts. We believe that this finding may be attributed to the significant effects of patient expectation, which is often ignored, on clinical scores. Satisfying patient expectations strongly correlates with the level of satisfaction expressed by patients after surgery. We feel that patient dissatisfaction, which could develop in a certain proportion of patients without a determinable cause, is due to the fact that the patients’ expectations prior to the operation were not satisfied after the operation. Therefore, we do not find it correct to determine the presence of internal rotation in patients with anterior knee pain and attribute such pain to this rotational difference. More research is needed on this subject.
